# The autophagy inducer trehalose stimulates macropinocytosis in NF1-deficient glioblastoma cells

**DOI:** 10.1186/s12935-022-02652-5

**Published:** 2022-07-21

**Authors:** Barbara Del Bello, Alessandra Gamberucci, Paola Marcolongo, Emilia Maellaro

**Affiliations:** Department of Molecular and Developmental Medicine, University of Siena, Via A. Moro, 53100 Siena, Italy

**Keywords:** Trehalose, Glioblastoma cells, Macropinocytosis, Methuosis, NF1, ERK 1/2, Autophagy

## Abstract

**Background:**

Glioblastoma is a highly aggressive brain tumor. A big effort is required to find novel molecules which can cross the blood–brain barrier and efficiently kill these tumor cells. In this perspective, trehalose (α-glucopyranosyl‐[1→1]‐α‐d‐glucopyranoside), found in various dietary sources and used as a safe nutrient supplement, attracted our attention for its pleiotropic effects against tumor cells.

**Methods:**

Human glioblastoma cell lines U373-MG and T98G were exposed to trehalose and analyzed at different time points. Cell proliferation was evaluated at medium term, and clonogenic capacity and cell morphology were evaluated at long term. Western blot was used to evaluate biochemical markers of autophagy (also measured in cells co-treated with EIPA or chloroquine), and mTOR, AMPK and ERK 1/2 signalling. Macropinocytosis was evaluated morphologically by bright-field microscopy; in cells loaded with the fluorescein-conjugated fluid-phase tracer dextran, macropinocytic vacuoles were also visualized by fluorescence microscopy, and the extent of macropinocytosis was quantified by flow cytometry.

**Results:**

The long-term effect of trehalose on U373-MG and T98G cell lines was impressive, as indicated by a dramatic reduction in clonogenic efficiency. Mechanistically, trehalose proved to be an efficient autophagy inducer in macropinocytosis-deficient T98G cells and an efficient inducer of macropinocytosis and eventual cell death by methuosis in U373-MG glioblastoma cells, proved to be poorly responsive to induction of autophagy. These two processes appeared to act in a mutually exclusive manner; indeed, co-treatment of U373-MG cells with the macropinocytosis inhibitor, EIPA, significantly increased the autophagic response. mTOR activation and AMPK inhibition occurred in a similar way in the two trehalose-treated cell lines. Interestingly, ERK 1/2 was activated only in macropinocytosis-proficient U373-MG cells harbouring loss-of-function mutations in the negative RAS regulator, NF1, suggesting a key role of RAS signalling.

**Conclusions:**

Our results indicate that trehalose is worthy of further study as a candidate molecule for glioblastoma therapy, due to its capacity to induce a sustained autophagic response, ultimately leading to loss of clonogenic potential, and more interestingly, to force macropinocytosis, eventually leading to cell death by methuosis, particularly in tumor cells with RAS hyperactivity. As a further anticancer strategy, stimulation of macropinocytosis may be exploited to increase intracellular delivery of anticancer drugs.

## Background

Glioblastoma multiforme (GBM) or grade IV astrocytoma (World Health Organization classification) accounts for more than half of all malignant brain tumors. It is highly aggressive, chemo-resistant and relapse-prone. The standard therapeutic approach, i.e., surgical removal if possible, followed by radiotherapy plus concomitant and adjuvant chemotherapy with temozolomide, elicits a very poor response in terms of survival rate. Moreover, the inability of many drugs to cross the blood–brain barrier (BBB) to access the tumor site is a prominent obstacle to the effectiveness of available and new chemotherapeutics [1]. Thus, there is a need for new molecules that can cross the BBB and efficiently target cell mechanisms for killing glioblastoma cells.

Macropinocytosis is a non-specific, clathrin-independent endocytic pathway through which a non-selective internalization of large portions of extracellular fluid occurs. To internalize extracellular material, polymerization of actin filaments is required to create characteristic membrane ruffling, which gives rise to endocytic vesicles termed macropinosomes (0.2–5 μm in diameter). Trafficking in the cytosol, macropinosomes can be recycled to the cell membrane or fused with lysosomes for degradation of their cargo, thus providing different nutrients to the cell [2]. Like autophagy, macropinocytosis allows cells to survive in nutrient-poor conditions. Unlike autophagy, by internalizing extracellular material, macropinocytosis increases net biomass and can fuel high metabolic demand. On the other hand, as in the case of cell death due to extensive autophagy, extreme and irreversible vacuolization due to macropinocytosis can cause a peculiar caspase-independent cell death process, termed methuosis, where vesicles accumulate in the cytoplasm and fuse with each other, forming very large vacuoles that eventually cause cell rupture [3].

Trehalose (α-glucopyranosyl‐[1→1]‐α‐d‐glucopyranoside) is a natural disaccharide of glucose, found in a variety of organisms, including plants, bacteria, fungi and insects. For human beings, the major natural dietary sources of trehalose are mushrooms, baker’s yeast and brewer’s yeast. Listed “generally recognized as safe” (GRAS) by the Food and Drug Administration in 2000, it is also commonly used as a supplement in the food industry. Initially used as a cryoprotectant for tissues and cells, over the last 15 years trehalose has attracted attention for its therapeutic potential in a variety of neurodegenerative diseases [4] and cancer [5].

In two melanoma cell lines which differ greatly in chemosensitivity and radiosensitivity, we recently showed [6] that trehalose inhibits short-term cell proliferation and, even more, the colony-forming capacity in the long term. It also enhances ionizing radiation‐ and temozolomide-induced cytotoxicity, even in resistant melanoma cells. Mechanistically, we demonstrated that trehalose is able to induce a strong autophagic response in chemo-/radio-sensitive cells, or a premature senescence response in resistant cells. Furthermore, no cytotoxicity was observed in normal human melanocytes treated with trehalose. On this basis and since trehalose is known to cross the blood–brain barrier [7], it appeared worthwhile studying its effects in brain tumor cells, such as glioblastoma cells.

Here we report our finding that trehalose has low and high efficacy as an autophagy inducer in U373-MG and T98G human glioblastoma cells, respectively. More interestingly, as a new process induced by trehalose, we demonstrate that it is an efficient inducer of macropinocytosis only in NF1-deficient U373-MG cells, which are poorly responsive to stimulation of autophagy, and that autophagy and macropinocytosis appear to be mutually exclusive events. In both cell lines, trehalose exerted an impressive reduction in clonogenic capacity in the long term: specifically, the remarkable, long-lasting macropinocytosis occurring in U373-MG cells eventually culminated in methuosis, a peculiar mode of cell death.

## Methods

### Cell cultures and treatments

The established human glioblastoma cell lines, U373-MG and T98G, originally provided by ECACC, were a kind gift from Prof. Sergio Comincini (University of Pavia). Both cell lines were cultured in RPMI-1640 (Sigma-Aldrich), containing 10% heat-inactivated fetal bovine serum (FBS) (Euroclone), 2mM glutamine (Sigma-Aldrich) and 50 mg/L gentamycin (Sigma-Aldrich), at 37 °C, in a humidified atmosphere with 5% CO_2_. Cell lines were confirmed negative for Mycoplasma contamination by periodic checks with the MycoAlert Mycoplasma detection kit (LTO7-218, Lonza Rockland).

Cells were routinely harvested by a brief incubation in 0.05% trypsin-0.02% EDTA solution (Sigma) and reseeded before reaching confluence. For experiments, seeded cells were rested overnight and then treated in fresh medium with 0–120 mM trehalose (Sigma), dissolved in complete medium, for different experimental times.

Cell proliferation was evaluated by counting the harvested cells in a Bürker chamber, and each sample at each treatment time was expressed as percentage of cell number at the start of the experiment. Cell viability was assessed by using the trypan blue exclusion assay.

In selected experiments aimed to evaluate the actual autophagic flux, cells were cotreated (for the final 6 h) with 30 µM chloroquine (Sigma), a lysosomotropic compound that raises the lysosomal pH thus inhibiting the fusion of autophagosomes with lysosomes [6].

In selected experiments for autophagy and macropinocytosis evaluation, cells were cotreated with 25 µM of the Na+/H+ exchanger inhibitor 5-(N-ethyl-N-isopropyl) amiloride (EIPA) (Sigma). In preliminary experiments, the selected EIPA concentration proved not to be cytotoxic, even at long exposure times.

### Microscopy analysis

To evaluate the morphological changes induced by trehalose, U373-MG and T98G cells were seeded in 12-well plates containing 13 mm glass coverslips, or in 4-well cell culture chamber with coverglass bottom (Sarstedt), at a density of 40 × 10^3^ cells/well. Cells were allowed to adhere overnight and then left untreated or treated with different concentrations of trehalose, dissolved in RPMI medium without phenol red.

#### Phase contrast microscopy

To determine the percentage of vacuolized cells, phase-contrast images of live cells were captured on a Nikon Eclipse Ti microscope, equipped with DS-Q1Mc camera and a NIS element software (Nikon). Five fields (each containing 40–200 cells) were randomly selected and photographed by using the 10× phase-contrast objective, Ph1. Cells containing one or more phase-lucent vesicles with diameter > 3 μm or containing more than 4 smaller vacuoles (1–3 μm of diameter) were counted as positive. In selected experiments, to confirm the macropinocytic nature of the vacuoles, cells were treated with EIPA, as above, and photographed by using a 10× phase-contrast objective.

Cell morphology at long-term treatment with trehalose was assessed in the 12-well plates used for the clonogenic assay; just before fixation and subsequent staining of colonies (see below), fields randomly selected were photographed by using a 10× phase-contrast objective.

#### Fluorescence microscopy

To further confirm the macropinocytotic origin of these vesicles, the fluid-phase tracer Dextran 70 KDa conjugated with fluorescein isothiocyanate (FITC-Dex) (46845, Sigma-Aldrich) was used. Together with or after trehalose treatment (as detailed in figure legends), U373-MG cells were incubated with 0.5 mg/ml of FITC-Dex dissolved in complete RPMI medium without phenol red. Intracellular acidic compartments were labelled by incubating live cells with Lysotracker Red DND-99 (Invitrogen), dissolved in phenol red-free RPMI at the final concentration of 75 nM. Cells were treated with trehalose and concomitantly labelled with FITC-Dex and Lysotracker. After 3 h of treatment, live cells were washed three times with cold phosphate-buffered saline (PBS), and images were immediately taken by fluorescence microscopy, with the above-mentioned Nikon Eclipse Ti, by using a 60× oil immersion objective.

In selected experiments aimed to evaluate the possible occurrence of apoptosis, cells were seeded on coverslips, treated with trehalose, incubated with FITC-Dex, and then fixed with paraformaldehyde (PFA) 4% in PBS pH 7.4, for 20 min in the dark, at room temperature (RT). In fixed cells, nuclei were stained for 20 min with 4′,6-diamidino-2-phenylindole (DAPI), at a final concentration 1 µg/ml in PBS). Cells were examined by using a 60× oil immersion objective.

### Flow cytometry

U373-MG cells were seeded in six-wells plates and allowed to attach overnight. Cells were treated with 90 mM trehalose in phenol red-free RPMI medium for 48 h, and FITC-Dex (final concentration 0.5 mg/ml) was added for the final 3 or 24 h. In selected experiments, to confirm that FITC-Dex uptake derived from a macropinocytic process, control and trehalose-treated cells were co-treated with 25 µM EIPA for the entire experimental time. At the end of incubation, FITC-Dex-containing medium was promptly removed, cells were washed three times with ice-cold-PBS, pH 7.4, and harvested by trypsinization. Cells were pelleted by centrifugation (1000*g* for 5 min), resuspended in ice-cold PBS-1% FBS at the final concentration of 200 × 10^3^/ml, and immediately analyzed. In each experiment, blank samples (cells without FITC-Dex) were also evaluated, giving no difference between control and treated cells. A minimum of 10,000 cells per sample was analysed by flow cytometry using a Guava EasyCyte 6-2 L cytometer (Luminex Corporation), and data were analysed with FlowJo software (Tree Star).

### Clonogenic assay

The clonogenic assay was performed essentially according to a well-established procedure [8]. Three hundred cells were seeded into 12-well plates, allowed to attach overnight, then treated with different doses of trehalose, and let to grow for about 2 weeks in the incubator. Cells were then washed with PBS, fixed with PFA (4% in PBS), and stained with 0.5% crystal violet dissolved 25% methanol. Colonies containing ≥ 50 cells were automatically counted using ImageJ software. The clonogenicity of treated cells was evaluated as percentage on the number of colonies in control cells. For the colony counting, the Clono-counter software was set up to count clones containing at least 50 cells, as commonly accepted.

### Western blot

At the end of experiments, cells detached by trypsinization were resuspended in ice-cold lysis buffer 20 mM HEPES–NaOH, pH 7.5, containing 10% glycerol, 0.1% CHAPS, 0.2% NP-40, 1 mM EDTA, 5 mM dithiothreitol (DTT), 1 mM phenylmethylsulphonyl fluoride (PMSF), protease inhibitor cocktail (Sigma-Aldrich), and phosphatase inhibitors (sodium orthovanadate 1 mM, sodium fluoride 10 mM and β-glycerophosphate 10 mM). After sonication on ice for 10 s (Vibracell, amplitude 60, 25 W) and centrifugation at 12,000*g* for 10 min at 4 °C, the supernatant of cell lysates was assayed for protein concentration by using the Bradford reagent (B6916, Sigma-Aldrich). Equal amounts (25 µg) of proteins were separated by sodium dodecyl sulphate polyacrylamide gel electrophoresis (SDS-PAGE) on 4–20% Mini-Protean Precast gels (Bio-Rad Laboratories) or home-made 15% polyacrylamide gel (for ERK 1/2 only), for 60 min at 140 V, and electrophoretically transferred to 0.22 μm nitrocellulose membranes (Bio-Rad Laboratories) for 90 min at 260 mA. Before adding primary antibodies, quality control and transfer efficiency were assessed by reversible Ponceau S (P7170, Sigma-Aldrich) membrane staining of total proteins. After destaining with 0.01 M NaOH and rinsing with deionized water, nitrocellulose membranes were blocked for 1 h at RT with 5% bovine serum albumin (BSA) in TBS (Tris-buffered saline) (50 mM Tris, 150 mM NaCl, pH 7.5)/0.05% Tween 20 (TBST), and probed overnight at 4 °C with the following primary antibodies: anti-LC3A/B (referred to as LC3) (dilution 1:1000; L7543, Sigma-Aldrich), anti-phospho-p70 S6 Kinase (Thr389) (dilution 1:800; 9234, Cell Signaling Technologies), anti-p70 S6 Kinase (dilution 1:1000; 2708, Cell Signaling Technologies), anti-phospho-AMPKα (Thr172) (dilution 1:1000; 2535, Cell Signaling Technologies), anti-AMPKα (dilution 1:1000; 5831, Cell Signaling Technologies), anti-phospho-ERK 1/2 (Thr202/Tyr204) (dilution 1:400, 12D4, Santa Cruz Biotechnology), and anti-ERK 1/2 (dilution 1:400, C9, Santa Cruz Biotechnology). All primary antibodies were diluted in PBS-0.05% Tween 20 (PBST) with 2% BSA. After four washes with TBST, membranes were incubated for 1 h at RT with horseradish peroxidase-conjugated goat secondary antibodies (anti-rabbit R4880, or anti-mouse A5420, both Sigma-Aldrich), diluted in PBST with 1% skimmed milk.

Proteins were visualized by chemiluminescence (Clarity Western ECL Substrate, Bio-Rad Laboratories) with a CCD camera gel documentation system (ChemiDoc™ XRS+, Bio-Rad Laboratories). The intensity of protein bands of interest was quantified using Image Lab software (Bio-Rad Laboratories) and normalized by using the total amount of protein of the relative lane, as obtained by Ponceau S membrane staining. For p70 S6 Kinase, AMPKα and ERK 1/2, after immunoblotting for detection of phosphorylated form membranes were stripped and reprobed for the expression of the total protein [9]; the ratio phosphorylated protein/total protein was expressed as fold-change over the ratio of the proper control sample at each time.

### Statistics

Results are presented as mean ± SE, and the statistical significance of the differences was determined by the Student’s *t* test. Where reference sample was set to 1 or 100, results were presented as the mean fold-change or percentage ± SE over reference sample, and the statistical significance was determined by the Confidence Interval (CI).

## Results

### Trehalose induced remarkable cytoplasmic vacuolization, demonstrated to be macropinocytosis, in U373-MG cells

**Fig. 1:**
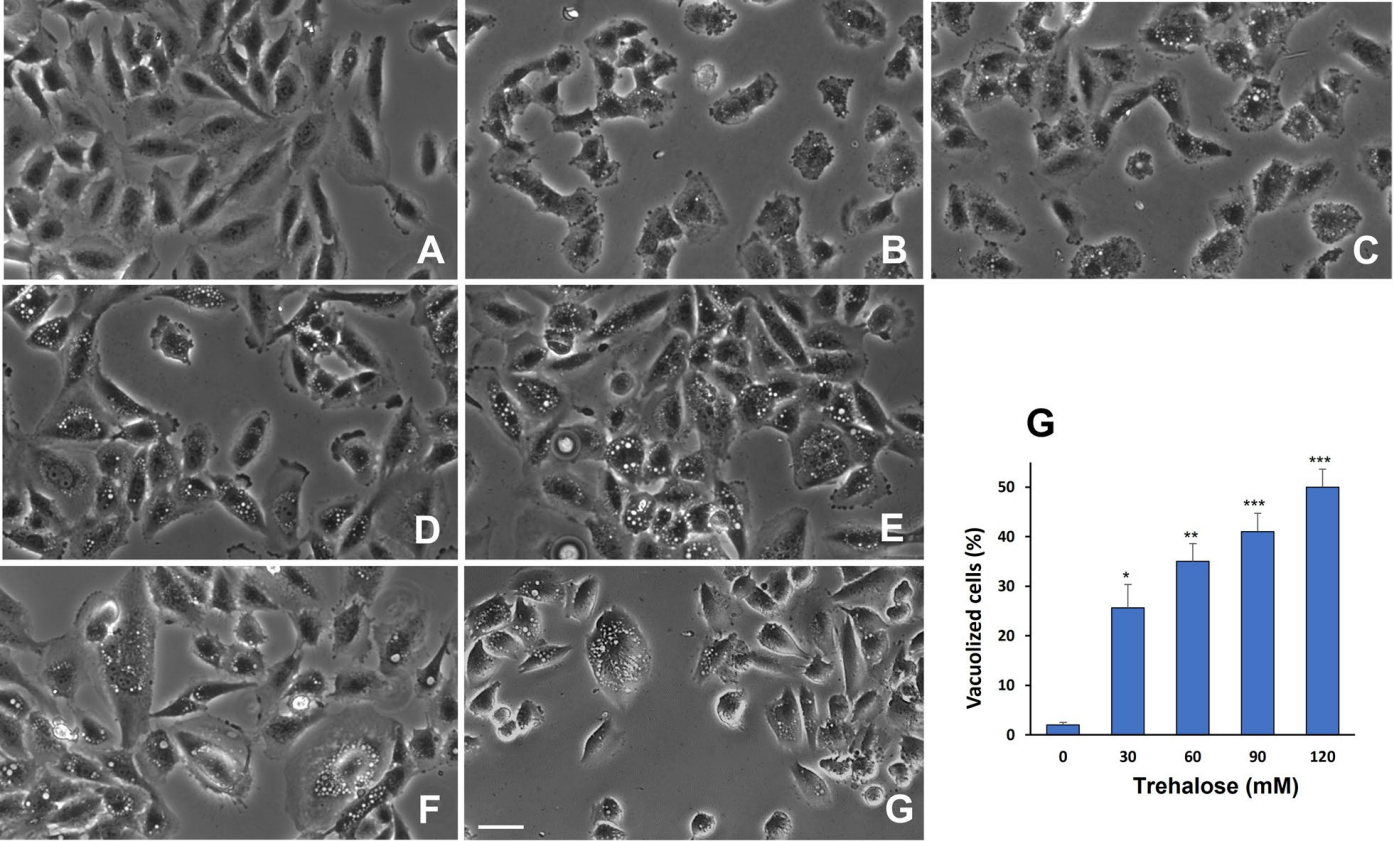
Trehalose induces cytoplasmic vacuolization in glioblastoma U373-MG cells. Phase-contrast microscopy images of live U373-MG cells left untreated (**A**) or treated for 48 h with trehalose 30 mM (**B**), 60 mM (**C**), 90 mM (**D**), or 120 mM (**E**). Cells treated with 90 mM trehalose for 72 h (**F**), or with 120 mM trehalose for 18 h (**G**). Scale bar = 50 mm. Percentage of vacuolized cells after 30–120 mM trehalose treatment for 48 h (**H**); cells containing one or more phase-lucent vesicles with diameter > 3 mm or containing more than 4 smaller vacuoles were counted as positive. Results are presented as mean ± SE of at least four independent experiments. *p < 0.05, **p < 0.01, and ***p < 0.001, significantly different from control cells

In U373-MG cells, trehalose (30–120 mM) induced dose-dependent cytoplasmic vacuolization after 48 h of treatment (Fig. 1B–E). Vacuolization continued beyond 48 h, as revealed at 72 h of treatment with 90 mM trehalose (Fig. 1F), and was already evident after 18 h of treatment with the higher doses of trehalose (Fig. 1G). With increasing doses of trehalose, a progressive increase in the number and size of highly refringent vacuoles appeared, as revealed by phase-contrast microscopy. The vacuoles that accumulated during the entire period of trehalose exposure were quite evenly distributed in the cytoplasm, from perinuclear to peripheral regions. Trehalose-treated cells showing larger and more numerous vesicles often appeared particularly swollen compared to control cells (Fig. 1A).

In order to quantify the formation of endocytic vesicles induced by 48 h of treatment with trehalose, the percentage of cells with cytoplasmic vacuoles was determined manually on phase-contrast microscopy images of live cells. Cells containing one or more phase-lucent vesicles with diameter > 3 μm or containing more than 4 smaller vacuoles (1–3 μm diameter) were counted as positive. As shown in Fig. 1H, the percentage of cells with phase-lucent vesicles increased in proportion to trehalose concentration.

**Fig. 2:**
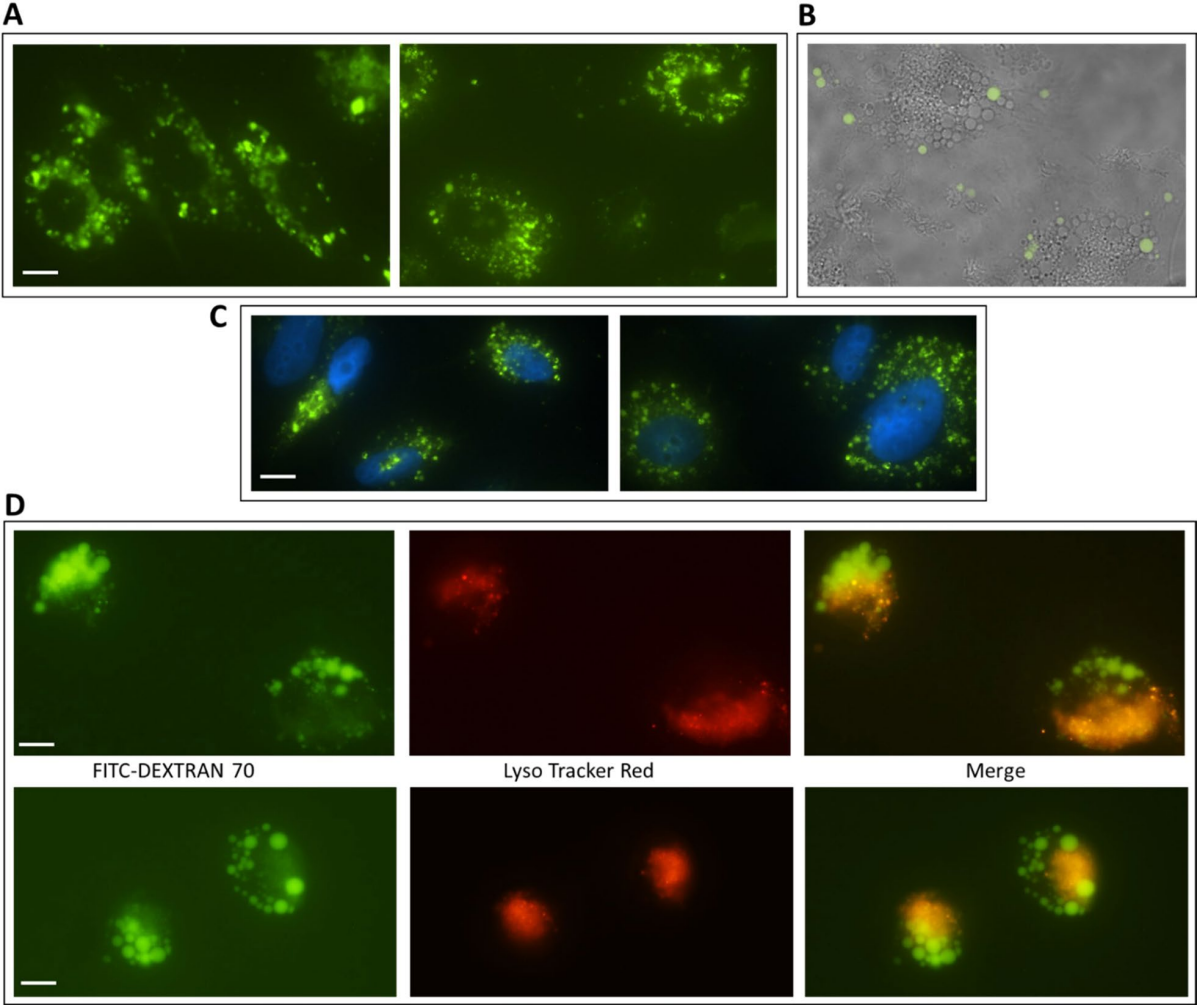
Trehalose induces macropinocytosis in U373-MG cells. Cells were treated with 90 mM trehalose for 48 h, and fluorescein-conjugated Dextran 70 kDa (FITC-Dex) was added at the final 24 h (**A**, **C**) or at the final 1 h (**B**); representative images of fixed cells observed by fluorescence microscopy (**A**), by fluorescence microscopy merged with bright-field illumination (**B**), or by fluorescence microscopy of DAPI-stained cells to visualize nuclei (**C**). **D** Cells were treated with 90 mM trehalose for 3 h, and concomitantly labelled with FITC-Dex and intracellular acidic compartment probe LysoTracker Red; representative images of live cells observed by fluorescence microscopy. Images derive from at least 3 independent experiments. Scale bar = 10 mm

The size of the vacuoles and their typical phase-lucent appearance suggested they were macropinosomes. To confirm this, we used the fluorescein-conjugated fluid-phase tracer, dextran 70 kDa (FITC-Dex) [10], which was added to the culture medium at different times for different trehalose concentrations and exposure times. Compared to lower molecular weight dextran, dextran 70 kDa is commonly used as a selective probe of macropinocytosis, since size limits its uptake into cells by small-scale endocytic processes such as clathrin-mediated endocytosis [11]. As shown in Fig. 2, many green-fluorescent vesicles (filled with FITC-Dex) of variable size were evident in cells treated with trehalose 90 mM. Figure 2 A shows cells treated for 48 h with trehalose and allowed to incorporate FITC-Dex for the last 24 h. The formation of macropinocytic vacuoles appeared to progress in time without slowing down; in fact, as shown in Fig. 2B, the green vacuoles (a small part of total vacuoles) were newly formed ones in trehalose-treated cells that had been allowed to incorporate the fluorescent tracer for just 1 h at the end of treatment.

In experiments where DAPI was used to label nuclei (Fig. 2C), neither chromatin condensation nor nuclear fragmentation were observed, and cell enlargement rather than shrinkage was frequently found. In addition to these morphological features, no caspase-3/-7 enzyme activity, evaluated as previously described by us [6], was detected even at 6 days of treatment (not shown), thus further ruling out that trehalose might induce canonical apoptosis.

To assess whether the macropinocytic vesicles fused with the lysosomal compartment and to reveal any possible overlap between macropinocytic vesicles (green) and lysosomes (red), we labelled lysosomes with Lysotracker Red. As shown in Fig. 2D, at a time of trehalose treatment as short as 3 h, the red-fluorescent cell space was distinctly separate from the regions containing medium to large vacuoles, and merge images showed that partial overlapping only occurred between smaller dextran-filled vacuoles and Lysotracker-positive structures. This suggests that the majority of macropinocytic vesicles were soon unable to fuse with lysosomes, fusing rather with each other to form larger structures, which eventually contributed to cell engulfment.

**Fig. 3:**
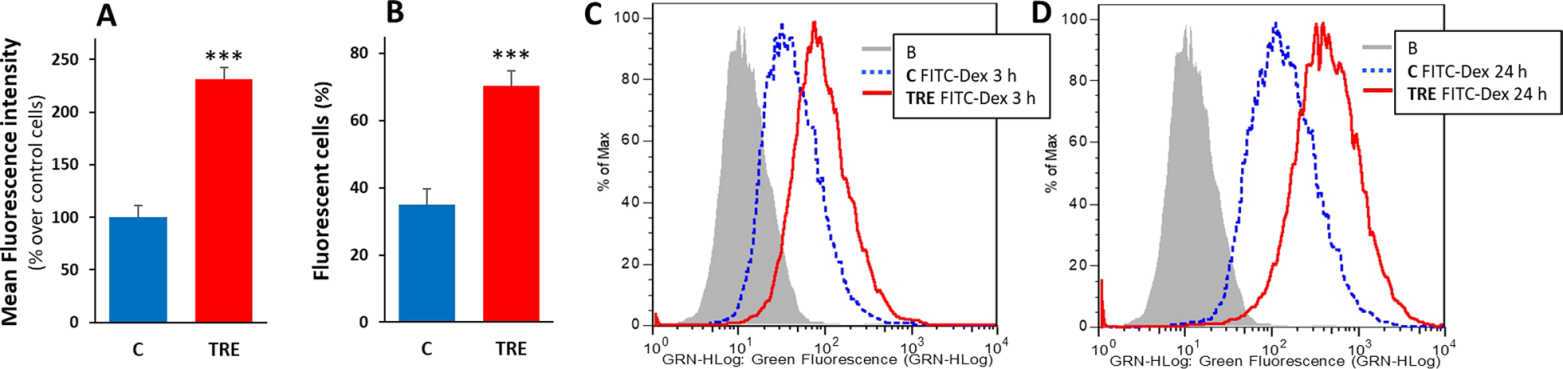
Quantitative evaluation of macropinocytosis by flow cytometry in U373-MG cells. Cells were untreated (C) or treated for 48 h with 90 mM trehalose (TRE), and let to incorporate FITC-dextran 70 kDa (FITC-Dex) for the last 3 h (**A**–**C**) or 24 h (**D**). The mean fluorescence intensity (**A**) and the percentage of fluorescent cells (**B**) were analyzed in at least 10,000 cells per sample. Results are presented as mean percentage (over control cells) ± SE (**A**) or mean ± SE (**B**) of 3–4 independent experiments performed in replicates. ***p < 0.001, significantly different from control cells. Representative histogram plots of fluorescence: FITC-Dex probe added for the last 3 h (**C**) or 24 h (**D**). B is blank sample (cells without probe)

Trehalose-induced macropinocytosis was also quantified by flow cytometry, evaluating incorporation of FITC-Dex. In these experiments, control cells and cells treated with 90 mM trehalose for 48 h were incubated with FITC-Dex for the last 3 h of treatment. As shown in Fig. 3, the uptake of the tracer was significantly higher in trehalose-treated cells than in control cells, both as mean fluorescence (reflecting the number and size of Dextran-labelled vesicles) (Fig. 3A, C) and as percentage of fluorescent cells (Fig. 3B). Taking the significant increase in these two parameters together, flow cytometry analysis confirmed that trehalose stimulated macropinocytosis in a remarkable way, as previously assessed by manual counting of vesiculated cells. Much higher fluorescence levels with very similar comparative results were obtained in cells allowed to take up the tracer for the final 24 h (Fig. 3D).

### Trehalose induced autophagy in U373-MG and T98G cells to a different extent

**Fig. 4:**
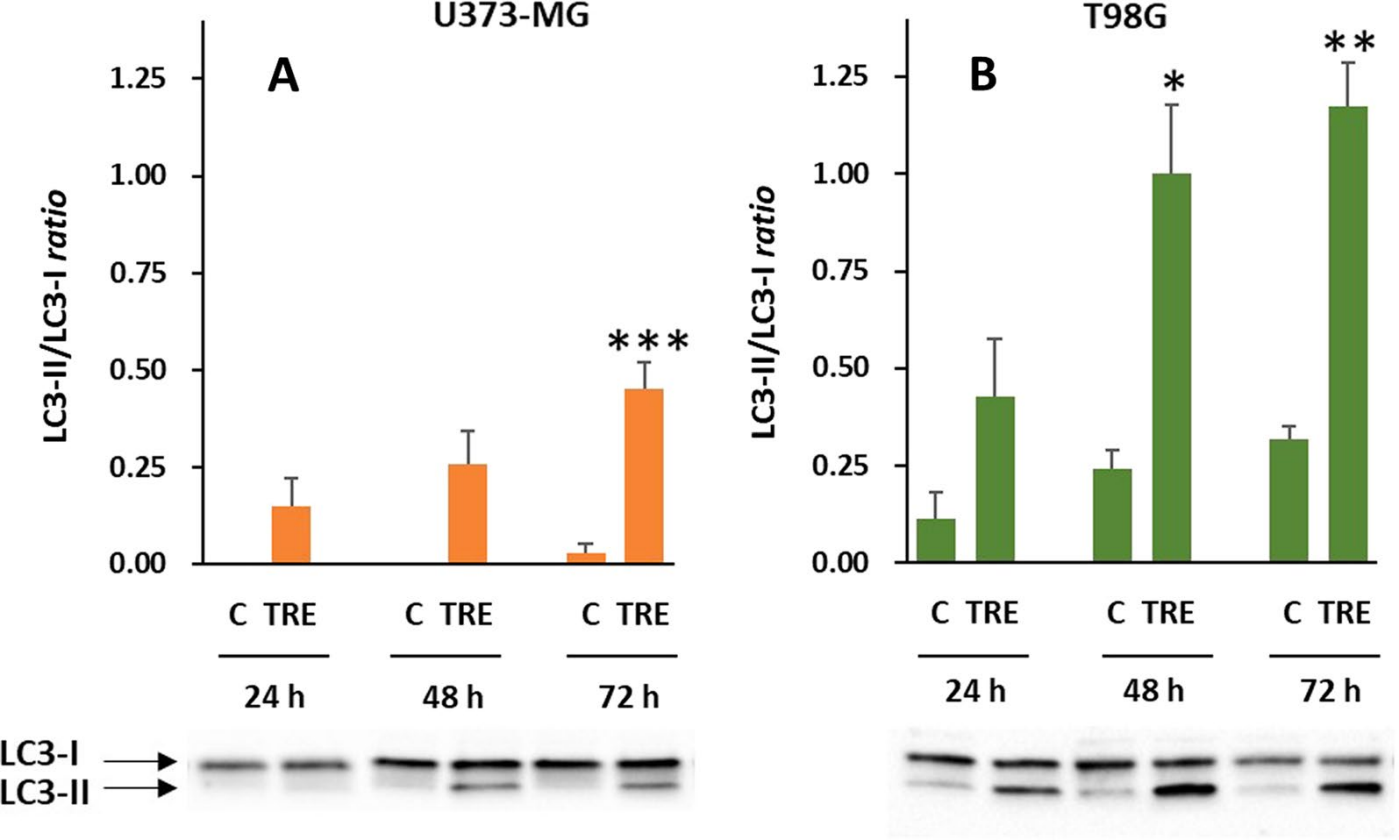
Trehalose induces autophagy to a different extent in glioblastoma U373-MG and T98G cells. U373-MG (**A**) and T98G (**B**) cells were untreated (C) or treated with 90 mM trehalose (TRE) for different experimental times. Autophagy was evaluated by the ratio LC3-II/LC3-I, which reliably evaluates the amount of autophagosomes. Results are presented as mean ± SE of 3 to 8 independent experiments (**A**), or 3 to 4 independent experiments (**B**). *p < 0.05, **p < 0.01, and ***p < 0.001, significantly different from control cells at the corresponding experimental time. Typical western blots are shown

**Fig. 5:**
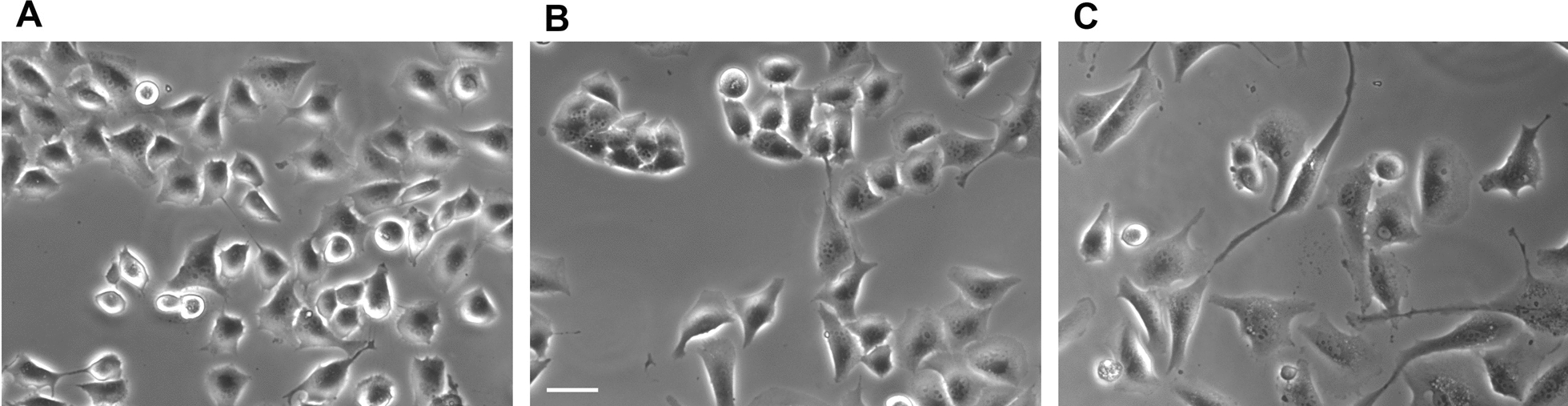
Trehalose does not induce vacuolization in T98G cells. Phase-contrast microscopy images of live T98G cells left untreated (**A**), treated for 72 h with trehalose 60 mM (**B**) or 120 mM (**C**). Images derive from 3 independent experiments. Scale bar = 50 mm

Alongside macropinocytosis, never hitherto described in trehalose-treated cells, this compound was also expected to stimulate macroautophagy (commonly referred to as autophagy) in U373-MG and T98G glioblastoma cells, as documented by us and others in a variety of normal and tumor cells [5, 6, 12]. Microtubule-associated protein 1 A/1B-light chain 3 (LC3) is the most widely used molecular marker of autophagosome biogenesis [13]. Native LC3 protein is first activated to LC3-I by Atg4-mediated proteolysis and then conjugated with phosphatidylethanolamine on the inner and outer autophagosome membranes, forming lipidated LC3-II; the binding remains throughout the pathway, and is crucial for elongation of the autophagosomal membranes and for assisting autophagosome maturation. Thus, the amount of LC3-II and the LC3-II/LC3-I ratio reliably quantify autophagosomes. In U373-MG cells, trehalose stimulated autophagy in a time-dependent manner, as evaluated by an increasing LC3-II/LC3-I ratio (Fig. 4A). However, when this response was compared with that stimulated by trehalose in T98G cells (in terms of LC3-II levels and LC3-II/LC3-I ratio) (Fig. 4B), U373-MG cells proved to have little propensity for autophagy, and this was also indicated by the almost undetectable basal levels of LC3-II in control cells. In a specular way, T98G glioblastoma cells, which proved to be autophagy-proficient, did not show phase-contrast microscope evidence of macropinocytosis in response to trehalose treatment (Fig. 5); even with higher trehalose concentrations and long treatment times such as 72 h, only a few cells showed a small number of very small vesicles.

### Trehalose-induced macropinocytosis appears to curb autophagy in U373-MG cells

**Fig. 6:**
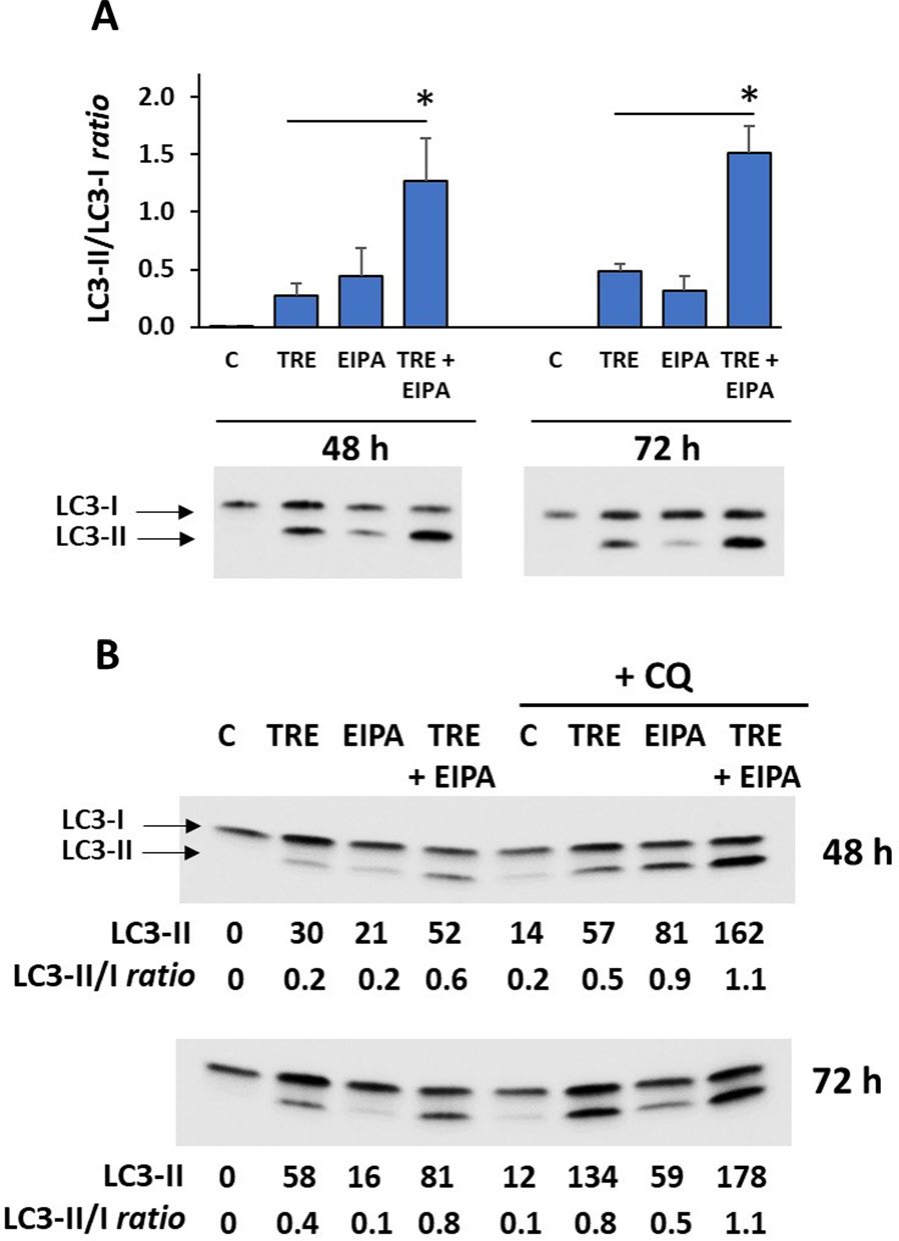
EIPA increases trehalose-induced autophagy in U373-MG cells. Cells were untreated (C), or treated with 25 mM EIPA (EIPA), 90 mM trehalose alone (TRE) or combined with EIPA (TRE + EIPA), for 48 or 72 h. **A** Autophagy was evaluated by western blot as the ratio LC3-II/LC3-I. Results are presented as mean ± SE of 4 to 7 independent experiments. *p < 0.05, significantly different from trehalose-treated cells. Typical western blots are shown. **B** Cells were cotreated with 30 mM chloroquine (CQ) for the final 6 h. Western blots derive from 2 independent experiments performed in duplicates, and numbers refer to the western blot above. Autophagy was evaluated as ratio LC3-II/LC3-I; LC3-II levels are also reported (as arbitrary units normalized by Ponceau S membrane staining)

Given the crosstalk between macropinocytosis and autophagy [14, 15], we wondered whether the low autophagic response induced by trehalose in U373-MG cells could somehow be correlated with its efficacy in stimulating macropinocytosis in these cells. To verify this, we co-treated U373-MG cells with the amiloride derivative 5-(N-ethyl-N-isopropyl) amiloride (EIPA). EIPA blocks a plasma membrane Na+/H + exchanger required for macropinocytosis and is regarded as a highly specific inhibitor of this process [16]. As shown in Fig. 6A, in terms of LC3-II and LC3-II/LC3-I ratio, co-treatment with 25 µM EIPA significantly increased the autophagic response induced by trehalose at 48 and 72 h of treatment, and the effect of combined molecules appeared to be synergistic. This EIPA-induced increase in autophagy was also seen in the absence of trehalose, suggesting that EIPA also affected the basal autophagic capacity of control cells.

To confirm that EIPA was actually increasing trehalose-induced autophagic flux and therefore to rule out that the higher levels of LC3-II resulted from its decreased lysosomal turnover, we used the established method, namely a final, short co-treatment with chloroquine (CQ), a lysosomotropic agent that inhibits the fusion of autophagosomes with lysosomes [6]. As shown in Fig. 6B, CQ co-treatment (for the final 6 h) further increased the LC3-II/LC3-I ratio and especially the levels of LC3-II, compared to cells not treated with CQ. Since this increase reflects the amount of LC3-II accumulating in autophagosomes, the results confirmed that EIPA was actually increasing the autophagic flux. Moreover, CQ further increased the LC3-II/LC3-I ratio and LC3-II levels in cells treated with trehalose but not EIPA, providing evidence that lysosomes are functional in cells undergoing macropinocytosis.

**Fig. 7:**
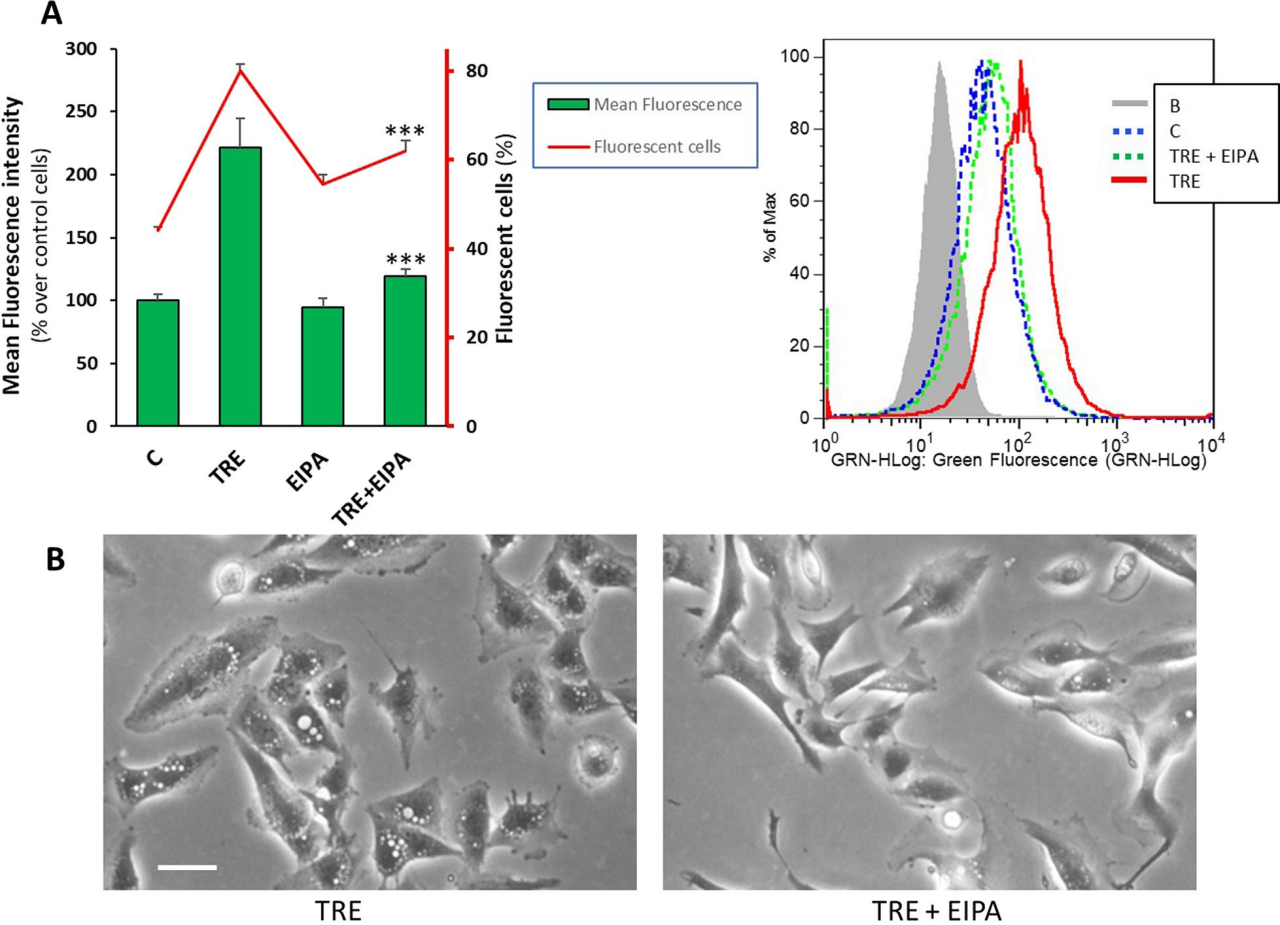
Treahalose-induced macropinocytosis in U373-MG cells is inhibited by EIPA. Cells were untreated (C), or treated for 48 h with 25 mM EIPA (EIPA), 90 mM trehalose alone (TRE) or combined with EIPA (TRE + EIPA); FITC-dextran 70 kDa (FITC-Dex) probe was added for the final 3 h. **A** The mean fluorescence intensity (columns) is reported as mean percentage (over control cells) ± SE, and the percentage of fluorescent cells (line) is reported as mean ± SE of 3 independent experiments performed in replicates. ***p < 0.001, significantly different from trehalose-treated cells. Representative histogram plots of FITC-Dex-labelled cells are shown. B is blank sample (cells without probe). **B** Phase-contrast microscopy images of live U373-MG cells treated with trehalose with or without EIPA. Images derive from 2 independent experiments. Scale bar = 50 mm

As expected, flow cytometry analysis confirmed that trehalose-induced macropinocytosis was effectively inhibited by EIPA (Fig. 7). In these experiments, control cells and cells treated with 90 mM trehalose for 48 h were co-treated with 25 µM EIPA (the same experimental condition as for autophagy assessment), and FITC-Dex was added for the final 3 h. In trehalose-EIPA co-treated cells, mean fluorescence fell to control level, and the percentage of fluorescent cells also decreased significantly (Fig. 7A). Consistently, a significant inhibition of macropinocytosis was also evident by phase-contrast microscopy (Fig. 7B).

### Trehalose-induced signalling

**Fig. 8:**
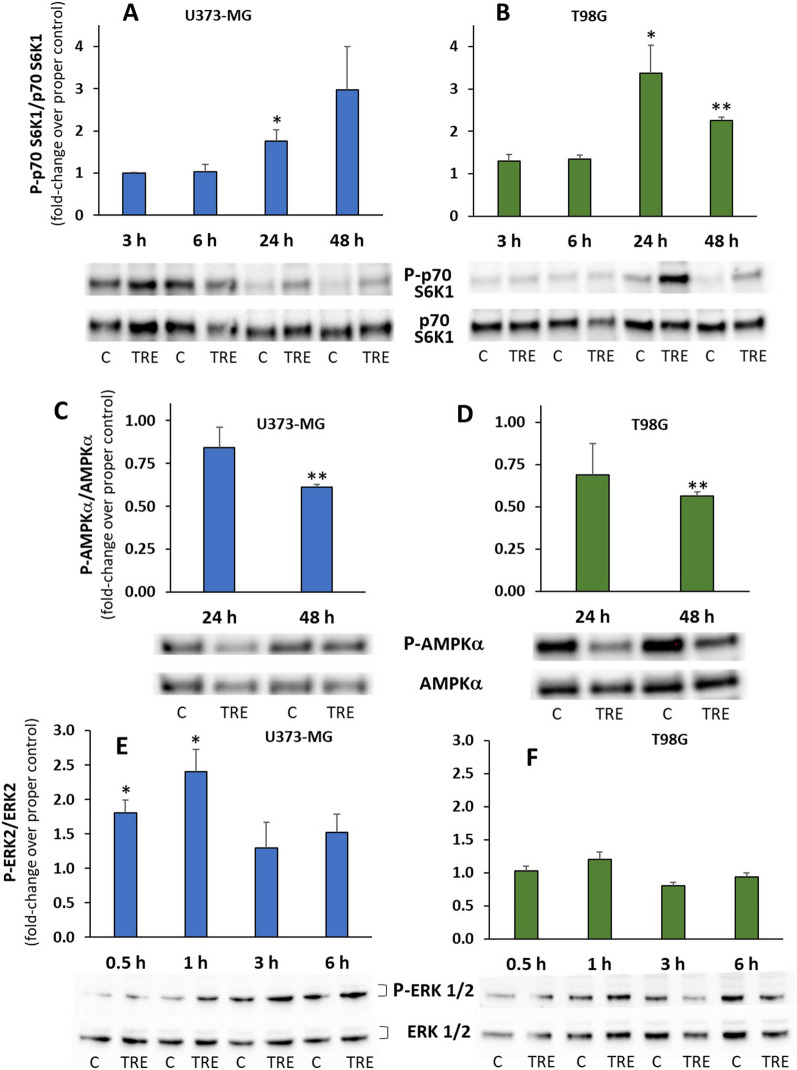
Trehalose modulates mTOR, AMPK and ERK1/2 activity. U373-MG (**A**, **C**, **E**) and T98G cells (**B**, **D**, **F**) were untreated (C) or treated with 90 mM trehalose (TRE) for different experimental times. mTOR activity (**A**, **B**) was evaluated as ratio between p70 S6K1 phosphorylated on Thr389 residue (P-p70) and total p70 S6K1 (p70); AMPK activity (**C**, **D**) was evaluated as ratio between the catalytic subunit AMPKa phosphorylated on Thr172 residue (P-AMPKa) and total AMPKa (AMPKa); ERK 1/2 activation (**E**, **F**) was evaluated as ratio between ERK2 phosphorylated on Thr202/Tyr204 residues (P-ERK) and total ERK2 (ERK2). For all of these proteins, after detection of the phosphorylated form, membranes were stripped and reprobed for the expression of the total protein. The ratio phosphorilated protein/total protein of trehalose-treated cells was expressed as fold-change over the ratio of the control sample at the corresponding experimental time. Results are presented as mean ± SE from 3 to 5 independent experiments. *p < 0.05, and **p < 0.01, significantly different from control cells at the corresponding time. Typical western blots are shown

A few regulatory mechanisms are reportedly shared by autophagy and macropinocytosis [14], including the regulation of mTORC1, i.e., complex 1 of mechanistic target of rapamycin (mTOR). Thus, in order to assess whether mTORC1 signalling was modulated differently in U373-MG and T98G cells, at different times of trehalose treatment we measured the phosphorylation status of the major downstream phosphorylation target of mTORC1, p70 S6K1 (70 kDa ribosomal protein S6 kinase 1); an increased ratio of p70 S6K1 phosphorylated on Thr389 threonine residue (P-p70) to total p70 S6K1 (p70) indicated activation of mTORC1. As shown in Fig. 8A, at short experimental times, no significant change in the P-p70/p70 ratio was observed in trehalose-treated U373-MG cells, whereas at longer times (24 and 48 h) the ratio was much higher in trehalose-treated cells. Indeed, in control cells the ratio tended to fall over time; thus, rather than stimulating an absolute increase in the P-p70/p70 ratio, the effect of trehalose seemed to spare or delay mTORC1 inhibition, presumably due to the greater availability of nutrients derived less from autophagy but mostly from macropinocytosis. Moreover, macropinocytosis is reported to directly activate mTORC1 [15]. While in control T98G cells the P-p70/p70 ratio was stable over time, an absolute increase in the ratio (suggestive of mTORC1 activation) was observed in trehalose-treated cells at 24 and 48 h (Fig. 8B), possibly due to recycling of basic anabolic components by the sustained autophagic process.

We also evaluated the activation status of AMP-activated protein kinase (AMPK) (Fig. 8C, D), a sensor of low glucose availability (and more generally low cell energy), able to inhibit mTORC1 activity [17]. AMPK activation was evaluated as the ratio of phosphorylation on Thr172 threonine residue in the catalytic α subunit (P-AMPKα), required for full activation of AMPKα, to total AMPKα. As expected and consistent with mTORC1 (re)activation, in the two glioblastoma cell lines significant inhibition of AMPKα was observed in trehalose-treated cells at the longest experimental time. As suggested above for mTORC1 up-regulation, AMPKα inhibition may also possibly be due to a more favourable energy balance afforded by trehalose (through stimulation of autophagy and/or macropinocytosis) with respect to control cells.

We also explored the possible role of RAS (hyper)activation in U373-MG cells demonstrated to undergo macropinocytosis after trehalose treatment, compared to macropinocytosis-deficient T98G cells. In our experimental model, we did not force any oncogenic expression of active *RAS*, and neither cell lines harbour any mutation in *RAS*, however only macropinocytosis-proficient U373-MG cells harbour frameshift indels in *NF1* [18]. The *NF1*-encoded protein neurofibromin-1 is a RAS GTPase-activating protein (GAP) that promotes hydrolysis of GTP to GDP, thus down-regulating RAS activity. The said loss-of-function mutations in NF1 [18], also confirmed by undetectable neurofibromin-1 expression in U373-MG cells [19], can therefore enhance RAS activation and subsequently promote downstream signalling, including the RAF-MERK-ERK pathway. Thus we measured ERK phosphorylation (on Thr202 and Tyr204 residues) in the two cell lines after short (0.5-6 h) treatment with trehalose, and we observed that the ratio of phosphorylated ERK (P-ERK) to total ERK (specifically ERK-2 isoform) only increased in U373-MG cells (up to 2.4-fold compared to the appropriate control), suggesting that trehalose triggers early activation of RAS in NF1-deficient cells (Fig. 8E, F).

### Cell proliferation and viability after trehalose treatment

**Fig. 9:**
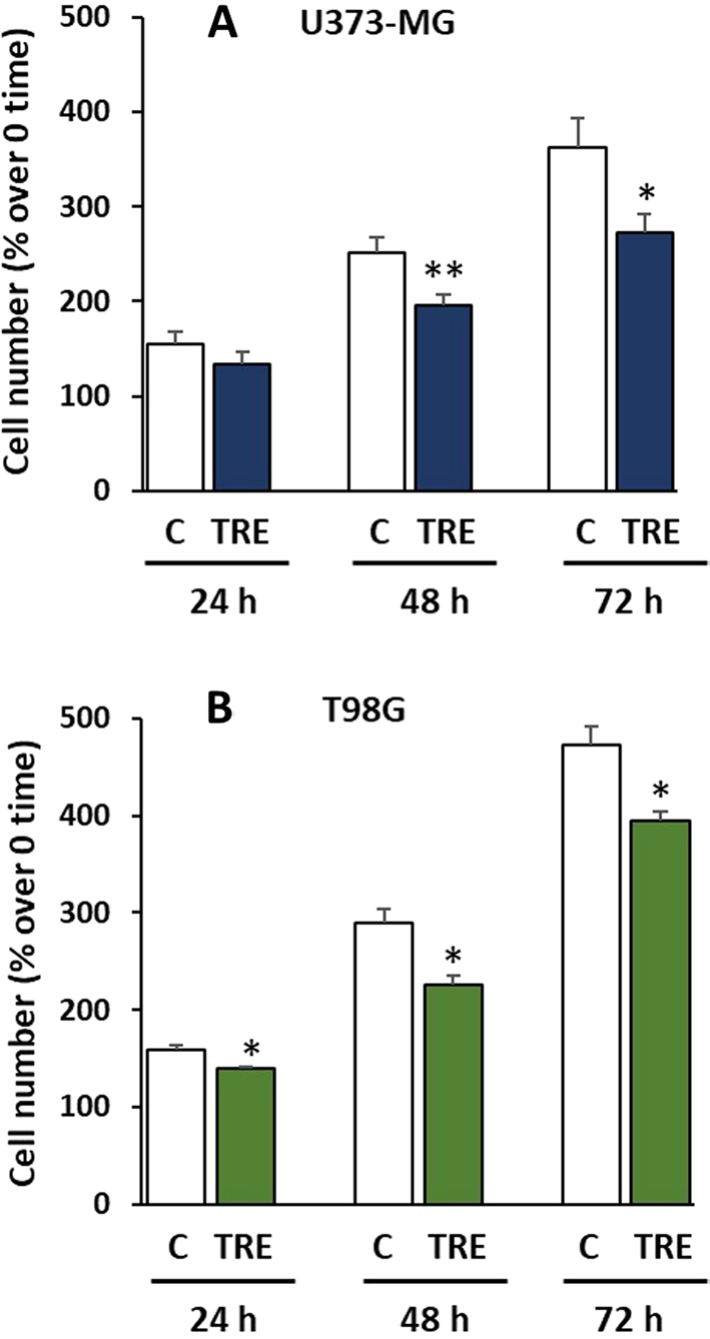
Trehalose inhibits cell proliferation. U373-MG and T98G cells were untreated (C) or treated with 90 mM trehalose (TRE) for different experimental times. Cell proliferation was expressed as percentage of cell number at the start of the experiment. **A** U373-MG cells; results are presented as mean ± SE from 8 independent experiments; **B** T98G cells; results are presented as mean ± SE from 3 independent experiments. *p < 0.05, and **p < 0.01, significantly different from control cells at the corresponding experimental time

To answer the question of the fate of U373-MG cells when remarkable macropinocytosis is induced by trehalose, we assessed cell growth and viability at 24–72 h of trehalose treatment. As shown in Fig. 9A, cell proliferation was lower than in control cells at 48 and 72 h of treatment. This limited cell growth was accompanied by minor cell detachment, however approximately 15% of adherent cells were Trypan Blue-positive at these experimental times, suggesting early loss of plasma membrane integrity. Trehalose treatment inhibited cell proliferation in T98G cells, without cell detachment or death (Fig. 9B).

**Fig. 10:**
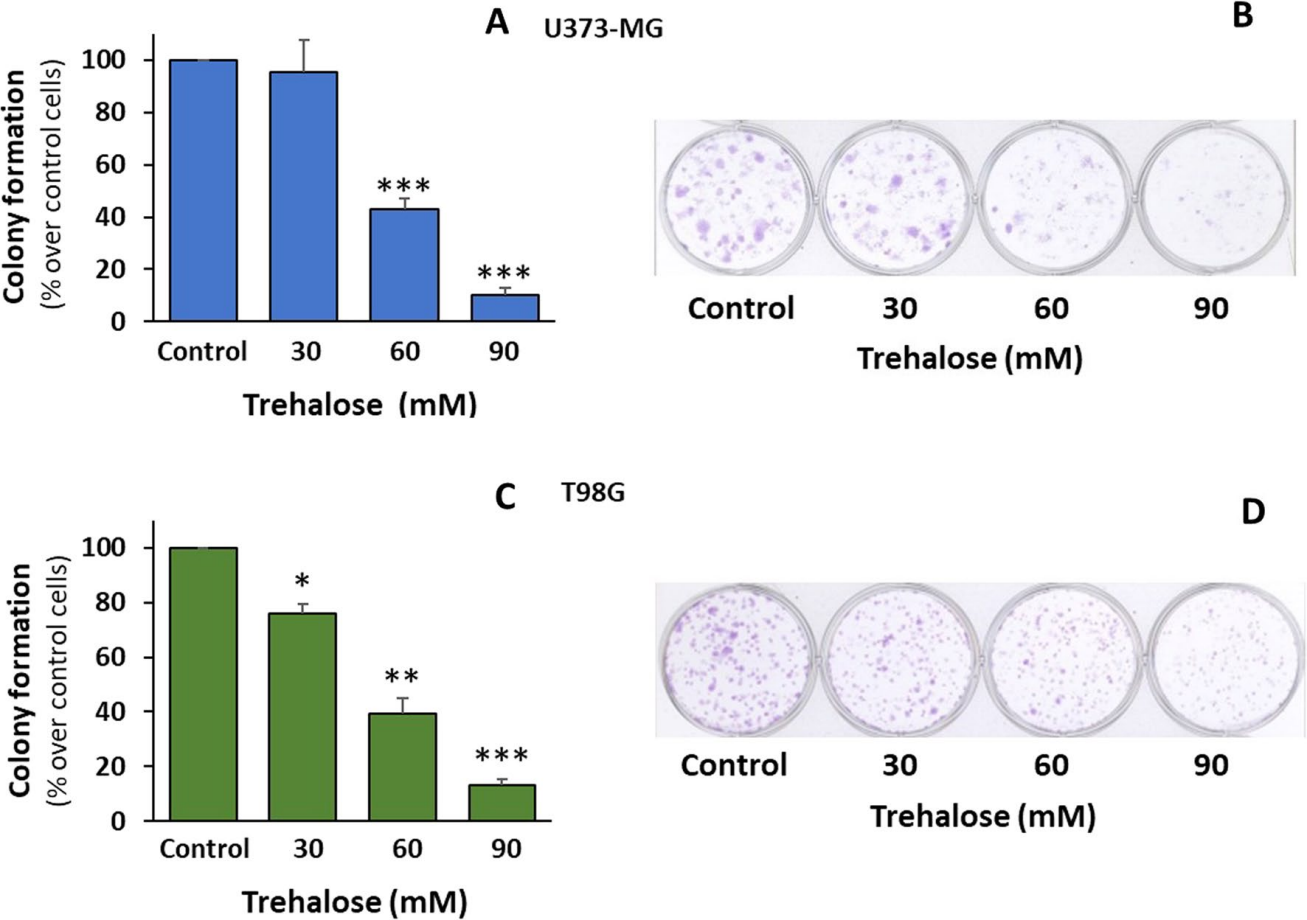
Trehalose induces a remarkable loss of clonogenic capability. U373-MG and T98G cells were untreated or treated with different concentrations of trehalose for 10–14 days. The clonogenic capability of U373-MG (**A**) and T98G cells (**C**) is reported as mean number of colonies (% over control cells) ± SE, from 5 or 3 independent experiments, respectively; *p < 0.05, **p < 0.01, and ***p < 0.001, significantly different from control cells. Representative plates are also shown (**B**, **D**), for U373-MG and T98G cells, respectively

Long-term cell fate (10–14 days) was also evaluated by clonogenic assay, which indicates the capacity of single cells (seeded at high dilutions) to form colonies (clones). Compared with the inhibition of cell proliferation occurring at the shorter treatment times, the long-term effect of trehalose on U373-MG cells was impressive (Fig. 10A, B), showing a significant dose-dependent reduction in clonogenic efficiency. Using the commonly accepted size parameter (i.e., counted clones contain at least 50 cells), the number of colonies in 30 mM trehalose proved to be almost the same as in control cells; indeed by eye, the total violet staining (total cells) of 30 mM trehalose wells was clearly less than that of untreated wells in all experiments. This was presumably due to the fact that in control sample the colonies partly merged, leading to a slight underestimation of colony number.

**Fig. 11:**
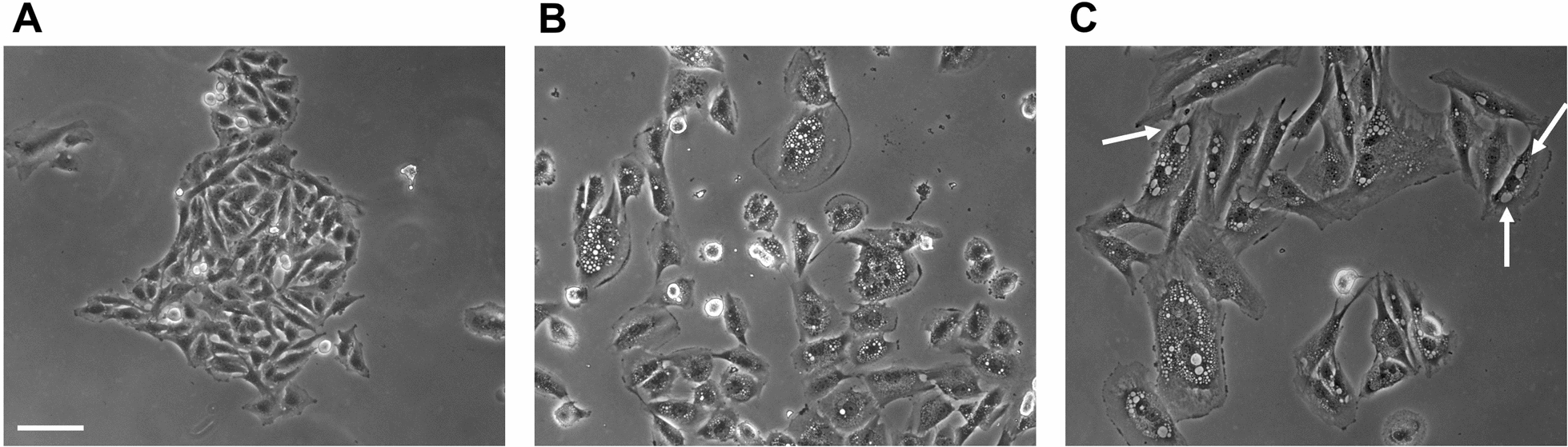
Trehalose induces cell death by methuosis in U373-MG cells. U373-MG cells were untreated (**A**) or treated with 90 mM trehalose for 10–14 days (**B**, **C**); phase-contrast microscopy images of live cells were obtained from the plates used for clonogenic assay (before fixation and staining). White arrows indicate less refringent large vacuoles, with dense material inside. Scale bar = 50 mm

At such long treatment times, the dramatic reduction in cell number was concomitant with methuosis (death by extensive macropinocytosis), as revealed by a remarkable deterioration in cell morphology (Fig. 11), particularly evident in isolated cells outside of colonies. Cells were generally swollen, and many showed flattened peripheral regions. Most cells contained many refringent vacuoles of various sizes which filled most of the cytoplasm (Fig. 11B, C), while other cells predominantly contained a few large vacuoles, suggesting end-stage coalescence of smaller vesicles; these larger vacuoles were usually less refringent and contained dense material (white arrows in Fig. 11C). There were also many rounded, detached, presumably dead cells. Also at these longer times, apoptotic cell death did not seem to occur, since dying cells showed intact nuclei, no chromatin condensation and well conserved nucleoli.

**Fig. 12:**
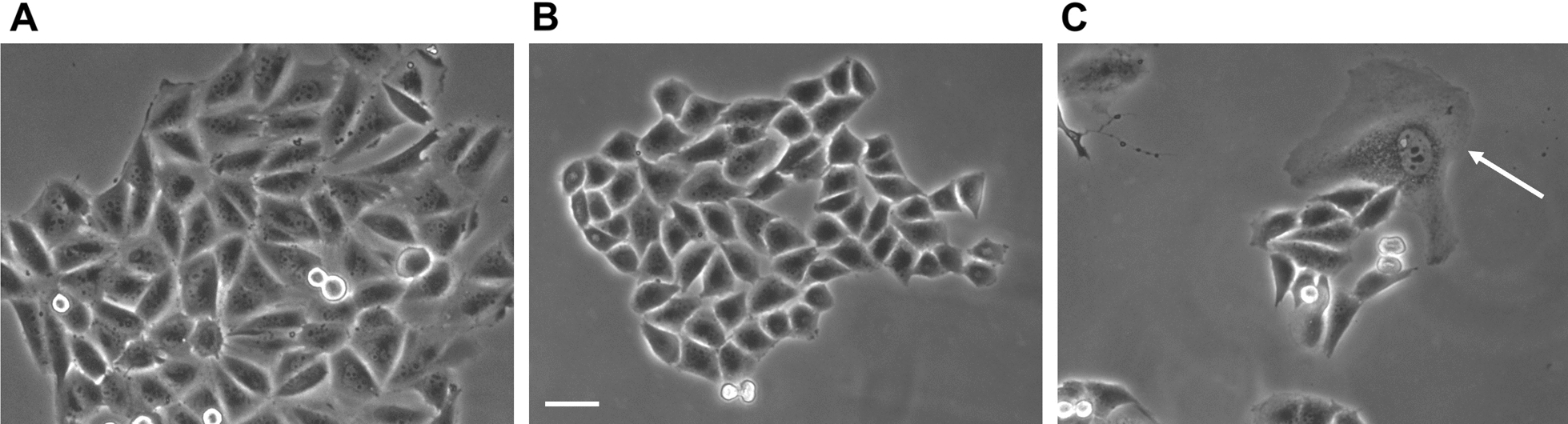
Trehalose does not induce methuosis in T98G cells. T98G cells were untreated (**A**) or treated with 90 mM trehalose for 10–14 days (**B**, **C**); phase-contrast microscopy images of live cells were obtained from the plates used for clonogenic assay (before fixation and staining). The white arrow indicates a damaged/dying cell. Scale bar = 50 mm

As in U373-MG cells, the clonogenic efficiency of T98G cells was dramatically and dose-dependently reduced in trehalose-treated cells (Fig. 10C, D). Their morphology did not show any signs of macropinocytosis even at these longer times (Fig. 12). As seen in A375 melanoma cells [6], such dramatic inhibition of clonogenic capacity was possibly due to the prolonged stimulation of autophagy, which eventually causes some form of autophagy-associated cell damage in a few cells. Indeed, treated cells generally appeared healthy, and isolated cells or those at the periphery of small colonies seemed swollen with many cytoplasmic granules. The number of rounded/detached cells was also smaller than in treated U375-MG cells.

## Discussion

There is an emerging need to find natural/alternative compounds with anticancer efficacy and adequate pharmacokinetic properties that do not affect the viability and physiological events of normal cells, thus minimising the systemic side-effects associated with current anti-cancer therapies. From this perspective, trehalose (a disaccharide of glucose monomers joined by a (1→1) glycosidic bond) [20] is attracting the attention of researchers due to its pleiotropic, protective role in a variety of pathophysiological conditions, as recently highlighted [21]. In cancer cell biology, under different experimental and cellular contexts, trehalose and its derivatives have been demonstrated to target various cell processes, including increased apoptosis, and decreased cell proliferation, migration and metastasis [5].

The best studied molecular pathway stimulated by trehalose is macroautophagy [21]. Among the different forms of autophagy (including macroautophagy, microautophagy and chaperone-mediated autophagy), macroautophagy (commonly referred to as autophagy) is a catabolic self-eating process through which unnecessary or dysfunctional cytoplasmic organelles and macromolecules are sequestered in double-membrane autophagosomes, which eventually fuse with lysosomes forming autophagolysosomes. In these vesicles, acid hydrolases break down the cargo, and the resulting metabolic components can be recycled [22]. This process conserved through evolution relies on several proteins (including those coded by AuTophagy-related Genes, ATGs) which are under close transcriptional and post-translational regulation, and act in a coordinated manner in different steps of autophagosome formation and maturation [23]. Autophagy is constitutively active at a basal level and can raise under various stress conditions, thus acting as a survival mechanism. However, in several pathophysiological settings, extensive activation of autophagy hampers cell recovery and culminates in growth arrest of cells and their eventual demise [24].

We recently showed [6] that trehalose inhibits short-term cell proliferation and especially long-term colony-forming capacity in two melanoma cell lines which differ greatly in chemosensitivity and radiosensitivity. It also enhances ionizing radiation‐ and temozolomide-induced cytotoxicity, even in resistant melanoma cells. Mechanistically, we demonstrated that trehalose an induced a strong autophagic response in chemo-/radio-sensitive cells, or a premature senescence response in resistant cells, whereas in normal melanocytes, it induced a minor autophagic response without affecting cell viability. Since trehalose crosses the blood–brain barrier [7], it seemed worthwhile investigating its effects on brain tumor cells, such as glioblastoma cells, and in fact we found that trehalose is an efficient autophagy inducer in T98G glioblastoma cells, and an efficient inducer of macropinocytosis and eventually cell death by methuosis in U373-MG glioblastoma cells, proved to respond poorly to stimulation of autophagy.

Macropinocytosis is a clathrin-independent form of endocytosis initiated by actin-dependent projections of the plasma membrane (lamellipodia), which envelop large portions of extracellular fluid [2, 10, 25]. Contact between membrane ruffles followed by membrane fission give rise to endocytic vesicles known as macropinosomes. These vesicles, which mature while being trafficked in the cytosol, can be recycled back to the plasma membrane or can fuse with lysosomes, in the latter case digesting the cargo and releasing basic components into the cytosol. By increasing net biomass, this process is particularly beneficial for tumor cells, particularly those harbouring activating mutations of *RAS* [26], fuelling anabolic pathways and proliferation under conditions of extracellular nutrient limitation. However, loss of cell viability (termed methuosis) may be caused by massive cytoplasmic vacuolization [27]: macropinosomes fuse with each other, forming many larger vacuoles which eventually cause rupture of the plasma membrane in a manner reminiscent of necrosis. This catastrophic cell event, as distinct from other forms of cell death, suggests an interesting therapeutic approach to killing tumor cells, especially those having intrinsic or acquired defects of the apoptotic machinery. Methuosis was first described in glioblastoma cells forced to overexpress a constitutively active form of RAS [27, 28]. Various stimuli, including a series of synthetic chalcones (indolyl-pyridinyl-propenones) [29, 30], plant-derived polyphenols [31] and CD99 immuno-targeting [32], have subsequently been demonstrated to trigger methuosis irrespective of activation of *RAS*.

Here we report that trehalose can induce macropinocytosis in U373-MG glioblastoma cells, a property never hitherto described among the pleiotropic effects of trehalose. The remarkable time- and dose-dependent cytoplasmic vacuolization induced by trehalose was evaluated by phase-contrast microscopy, which showed highly refringent vesicles increasing in number and diameter (ranging from 0.2 to 5 μm). The macropinocytotic origin of the vacuoles was confirmed by the capacity of these vesicles to incorporate the fluid-phase tracer, 70 kDa-dextran. Exploiting this uptake of fluorescein-labelled dextran, the extent of macropinocytosis was also quantified by flow cytometry analysis. Unlike in U373-MG glioblastoma cells, trehalose does not induce macropinocytosis in T98G glioblastoma cells, even with high doses and longer treatment times. The remarkable macropinocytosis induced by trehalose in U373-MG cells was accompanied by moderate inhibition of cell proliferation until 96 h of treatment, when loss of plasma membrane integrity was observed in almost 15% of adherent cells and cell detachment was still minor. By contrast, colony-forming capacity decreased dramatically in the long term, and did so in a dose-dependent manner. Interestingly, cell morphology clearly showed accumulation and fusion of unprocessed macropinosomes (i.e., not fused with lysosomes), leading to cell alterations typical of cell death by methuosis, which was particularly evident in isolated cells outside of colonies. Under medium- and long-term trehalose treatment, no morphological features of apoptosis (chromatin condensation, nuclear fragmentation, cell shrinkage) or the typical biochemical marker of apoptosis (caspase-3/-7 enzyme activity) were observed.

As widely reported in a variety of normal and tumor cells, we also found that trehalose stimulated autophagy in T98G and U373-MG glioblastoma cell lines, though macropinocytosis-proficient U373-MG cells appeared to be much less prone to autophagy. Although not yet fully deciphered, a crosstalk and a balance between autophagy and macropinocytosis appears to exist; they share a few signalling pathways, and both autophagic and macropinocytic vesicles eventually interact with the lysosomal compartment. In trehalose treatment of macropinocytosis-proficient U373-MG cells and autophagy-proficient T98G cells, these two processes appeared to act in a mutually exclusive manner. In fact, while inhibiting the macropinocytic process, co-treatment of U373-MG cells with EIPA significantly increased the autophagic response induced by trehalose.

Among the regulatory mechanisms shared by autophagy and macropinocytosis [14, 15], a key role is played by mTORC1, complex 1 of the mechanistic target of rapamycin (mTOR), a serine/threonine kinase involved in the stimulation of cell anabolic processes and the inhibition of catabolic processes in response to growth factors and nutrient availability [33]. mTORC1 activity (as evaluated by phosphorylation of its major target, p70S6K1) showed essentially the same behaviour in trehalose-treated U373-MG and T98G cells, being elevated at 24–48 h of treatment. Although control cells did not suffer severe starvation, even at longer experimental times, mTORC1 activation may be due to the fact that more nutrients derived from trehalose-induced autophagy are available in T98G cells. Likewise, in macropinocytosis-proficient U373-MG cells, mTORC1 (re)activation could be related to the anabolic advantage derived to a minor extent from autophagy but mainly from macropinocytosis. Moreover, macropinocytosis is reported to directly activate mTORC1 [15], which may contribute to down-regulation of autophagy. AMPK, a sensor of low glucose availability (and low cell energy in general), can inhibit mTORC1 activity through phosphorylation of the negative regulator TSC2 and the Raptor subunit [34]. AMPK activity, evaluated as phosphorylation of the catalytic α subunit, was inhibited to a similar degree in trehalose-treated samples of both cell lines, when mTORC1 was (re)activated. This is consistent with better anabolic status, not requiring AMPK-mediated inhibition of mTORC1.

As strictly regards autophagy stimulation, in both cell lines the canonical mTOR inhibition and AMPK activation did not occur, regardless of the extent of autophagy. Thus, we can assert that trehalose-induced autophagy is independent of mTOR inhibition (confirming a general consensus in the literature) and independent of AMPK activation.

The first evidence of macropinocytosis in glioblastoma cells was obtained in cells forced to express activated H-RAS [27]. The Rac1-dependent signalling pathway [27] and accumulation of vacuolar ATPase in the plasma membrane [35] were identified as essential regulators of macropinocytosis induced by aberrant RAS signalling. With this key role of RAS in macropinocytosis in mind, we also explored the possible role of RAS (hyper)activation in U373-MG cells undergoing macropinocytosis after trehalose treatment. In our experimental model, we did not force any oncogenic expression of active *RAS*, and *RAS* is not mutated in the glioblastoma cells used [18], in line with the low frequency of *H-RAS*, *K-RAS* and *N-RAS* mutations in glioblastoma patients [36, 37]. Interestingly, macropinocytosis-proficient U373-MG cells harbour frameshift indels in the *NF1* gene [18], and mutations or deletions in *NF1* are typical genetic abnormalities occurring in the transition from the proneural to the mesenchymal subtype of glioblastoma, which has the most aggressive malignant behaviour [38]. The protein encoded by *NF1*, neurofibromin-1, is a GTPase-activating protein (GAP) that negatively regulates the activity of multiple members of the RAS family by accelerating the hydrolysis of active GTP-RAS [39]. Loss-of-function mutations described in NF1, also confirmed by undetectable neurofibromin-1 expression in U373-MG cells [19], can therefore enhance RAS activity and subsequently promote downstream signalling, including the RAF-MERK-ERK pathway. In fact, trehalose treatment soon increases ERK phosphorylation, indicating that trehalose triggers early RAS activation in NF1-deficient U373-MG cells, and such signalling can be responsible for the subsequent stimulation of macropinocytosis. Consistently, NF1 deficiency on its own has been shown to increase macropinocytosis in *Dictyostelium* [40] and in macrophages [41].

Macropinocytosis did not occur in trehalose-treated T98G cells, although clonogenicity fell dramatically in a dose-dependent manner, presumably due to sustained stimulation of autophagy leading to inhibition of cell proliferation and eventually to autophagy-related cell damage. The role of autophagy in tumorigenesis and anticancer therapy is still controversial [42], however much evidence from tumor cells in various experimental settings suggests that exogenously forced autophagy is a general tumor-suppressive process, particularly crucial in cancer cells with impaired apoptotic mechanisms. Stimuli leading to extensive and long-lasting autophagy can block cell growth and eventually kill cells, also enhancing the efficacy of chemotherapy and radiotherapy, as we previously demonstrated in human melanoma cells treated with trehalose [6]. As regards the pharmacological treatment of glioblastoma, which is intrinsically resistant to apoptotic cell death [43, 44], increasing evidence indicates that induction of lethal autophagy has great potential [45]. In particular, stimulation of autophagy was found to counteract the oncogenic properties of glioblastoma stem cells [46] by inhibiting their proliferation, stemness, migration and chemo-/radio-resistance, while inducing their differentiation.

In conclusion, the capacity of trehalose to induce a sustained autophagic response in T98G glioblastoma cells, ultimately leading to loss of clonogenic potential, confirms that it is worthwhile studying molecules able to force autophagy with a view to their application in glioblastoma treatment. More interestingly, the capacity of trehalose to force macropinocytosis in NF1-deficient U373-MG glioblastoma cells is a novelty worth studying particularly in tumor cell types which are naturally prone to macropinocytosis, such as tumor cells with RAS hyperactivity due to oncogenic mutations in RAS itself or to loss-of-function of negative regulators of RAS. Cell death by methuosis, which eventually occurs after hyperstimulation of macropinocytosis, may be an alternative strategy for killing tumor cells, particularly those resistant to other types of cell death. For both purposes (detrimental autophagy or methuosis), trehalose virtually meets two requirements: (i) it crosses the blood–brain barrier, a necessity for treating primary brain tumors such as glioblastoma; and (ii) it does not show any systemic side-effects in animal models. In fact, this sugar is a common dietary component of natural and processed foods and is considered safe. Furthermore, as an added benefit compared to autophagy, macropinocytosis could be exploited to increase intracellular delivery of hydrophilic anticancer drugs, vesicular-formulated therapeutics, or targeted antibody-drug conjugates [47–49].

## Data Availability

Not applicable.

## Abbreviations

GBM: Glioblastoma multiforme
BBB: Blood–brain barrier
mTOR: Mechanistic target of rapamycin
mTORC1: Complex 1 of mechanistic target of rapamycin
p70 S6K1: 70 kDa ribosomal protein S6 kinase 1
AMPK: AMP-activated protein kinase
NF-1: Neurofibromin-1
ERK: Extracellular signal-regulated kinases
EIPA: 5-(N-ethyl-N-isopropyl) amiloride
FITC-Dex: Dextran 70 KDa conjugated with fluorescein isothiocyanate
PFA: Paraformaldehyde
DAPI: 4′,6-Diamidino-2-phenylindole
